# Applying software-defined networking to support telemedicine health consultation during and post Covid-19 era

**DOI:** 10.1007/s12553-020-00502-w

**Published:** 2020-11-02

**Authors:** Bokolo Anthony Jnr., Livinus Obiora Nweke, Mohammed A. Al-Sharafi

**Affiliations:** 1 Department of Computer Science, Norwegian University of Science and Technology, NTNU, NO-7491 Trondheim, Norway; 2 Information Security and Communication Technology, Norwegian University of Science and Technology (NTNU), Gjøvik, Norway; 3 Faculty of Computing, College of Computing and Applied Sciences, Universiti Malaysia Pahang, 26300 Gambang, Malaysia

**Keywords:** Medical systems, Telemedicine, Health consultation, Software-defined networking, COVID-19, Pandemic

## Abstract

The novel coronavirus disease-19 (COVID-19) infection has altered the society, economy, and entire healthcare system. Whilst this pandemic has presented the healthcare system with unprecedented challenges, it has rapidly promoted the adoption of telemedicine to deliver healthcare at a distance. Telemedicine is the use of Information and Communication Technology (ICT) for collecting, organizing, storing, retrieving, and exchanging medical information. But it is faced with the limitations of conventional IP-based protocols which makes it challenging to provide Quality of Service (QoS) for telemedicine due to issues arising from network congestion. Likewise, medical professionals adopting telemedicine are affected with low QoS during health consultations with outpatients due to increased internet usage. Therefore, this study proposes a Software-Defined Networking (SDN) based telemedicine architecture to provide QoS during telemedicine health consultations. This study utilizes secondary data from existing research works in the literature to provide a roadmap for the application of SDN to improve QoS in telemedicine during and after the COVID-19 pandemic. Findings from this study present a practical approach for applying SDN in telemedicine to provide appropriate bandwidth and facilitate real time transmission of medical data.

## Introduction

The COVID-19 pandemic has placed huge pressures on the healthcare system. Patients are required to stay at home in order to reduce the spread of the virus [1]. With social distancing being of extreme importance during this pandemic, much of healthcare has been move online or via telephone [2]. Due to the COVID-19 health crises several public health measures have been put in place such as temporal closing of public spaces and stay-at-home orders have been implemented across the world to support social distancing [3]. As access to diagnostic tools and healthcare is limited due to fewer availabilities of Personal Protective Equipment (PPE), Intensive Care Unit (ICU) beds, ventilators, etc.[4]. Efforts to decrease the spread of the virus and flatten the peak surge have led to extensive measures to reorganize and reallocate available resources. Including limiting or temporarily eliminating in-person health consultation [5]. Accordingly, technologies such as telemedicine is being integrated into health care delivery [2]. Over the years, telemedicine has been adopted due to its usefulness in providing medical services remotely [2].

Telemedicine which originated from the Greek word “far” [6], comprises using telecommunication networks to connect the physician and patient from a remote location [7]. Telemedicine in the form of email, video or audio consultation with patients, can significantly improve access to healthcare. Thus, medical professionals are moving their appointments online to provide healthcare to patients [5, 8]. Telemedicine can be deployed in real-time or synchronous audio and video communications between a physician and patient. It can also be deployed asynchronous via text messages, email, or by remote monitoring of patient medical data such as images [9]. Telemedicine provides remote assessment, diagnosis, treatment, and monitoring of patients [5]. Telemedicine decreases healthcare costs and facilitates health education of patients [9]. Findings from Elbeddini and Yeats [3] suggest that telemedicine provides a resource-effective approach to deliver pharmacist services in enhancing patient care.

The continuous growth and development of medical technology has led to in constant demand for better quality healthcare systems [10, 11]. This is particularly true for telemedicine which provides remote treatment and monitoring patients health anytime and anywhere [12]. However, the increase of internet users during the COVID-19 pandemic has require significant bandwidth, particularly during synchronous real-time health consultation. This has resulted to issues arising from network congestion [13]. Likewise, the COVID-19 pandemic currently has a huge impact on internet usage due to the work from home structure resulting to maximum usage of internet. As, there is need to transmit high quality video as well as provide suitable and fast communication path between the physician and patient [13]. Therefore, the aim of this study is to apply Software Defined Network (SDN) as a network paradigm to support Quality of Service (QoS) in telemedicine health consultations. SDN is an approach which supports network management and facilitates efficient network configurations. SDN decouples the control plane from the data plane [14].

SDN deploys a centralized network intelligence by separating the control plane (controlling process) from the data plane (forwarding process), which enables the management of the devices on the network from a central location [10, 13, 14]. Findings from this study propose the application of SDN for QoS improvement in telemedicine usage. The findings suggest that SDN specifies and monitors how traffic should be routed, providing lower delays and balancing. SDN provides better performance for telemedical health consultations. The reminder of the article is structured as Sect. 2 is methodology, Sect. 3 is findings. Section 4 is discussion and implications, and Sect. 5 is conclusion.

## Methodology

This study adopts a rapid literature review and secondary data was utilized to provide empirical evidence on software-defined networking, telemedicine, health consultation during COVID-19 pandemic similar to prior studies [12]. Therefore, the research question, review protocol and search procedure, and inclusion and exclusion criteria are discussed below;

### Research Questions

The following research questions to be addressed in this study include:

- **RQ1**: What is the significance of telemedicine health consultation in managing COVID-19 pandemic?
- **RQ2**: What are the issues that impact the deployment of telemedicine health consultation?
- **RQ3**: What recommendations can be employed to improve the deployment of telemedicine health consultation?
- **RQ4**: How can quality of service be improved during the deployment of telemedicine health consultation?

### Review Protocol and Search Procedure

Accordingly, we carried out a rapid review of prior studies published from 2017 till 2020 on different scientific libraries and databases which included Google Scholar, Sage, ScienceDirect, Inderscience Online, IEEE Xplore, Wiley, Springers, Emerald, Taylor & Francis, Scopus, PubMed, and Web of Science. The scientific libraries and databases utilized include the largest research information in multidisciplinary areas such as medicine, social science, health science, computer science, etc. The scientific libraries and databases allow searching, retrieving, and accessing of journals and conference proceeding articles. In conducting the online search from the scientific libraries and databases, the following key terms were employed: software-defined networking OR telemedicine OR QoS OR Quality of Service AND (software-defined networking OR telehealth), Coronavirus 2019 OR COVID-19 AND (SDN OR telemedicine) AND (SDN OR telehealth), in English.

**Fig. 1:**
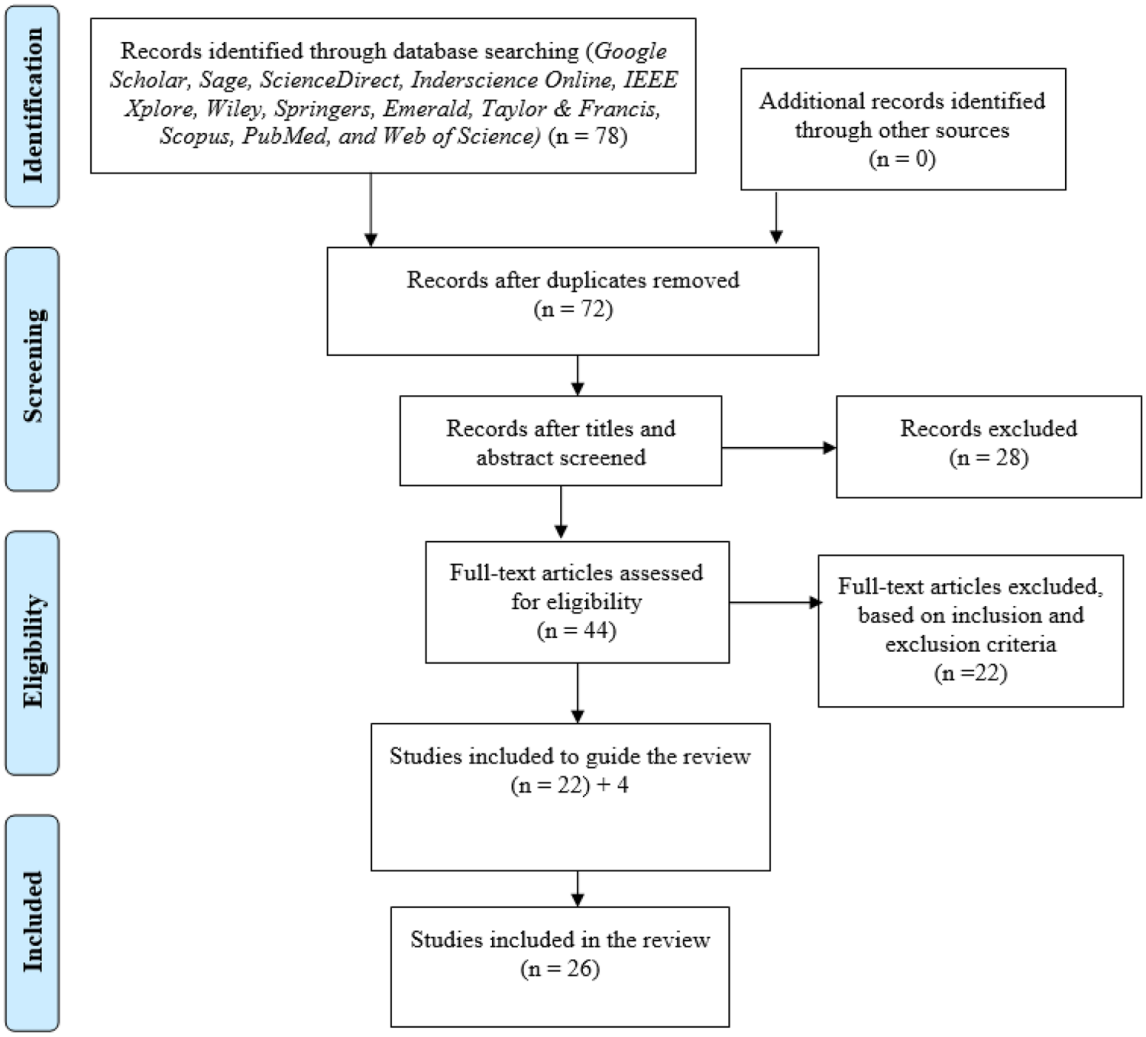
PRISMA flowchart for literature search process

The search results retrieved 78 articles using the above mentioned keywords. 6 papers were found as duplicates and were removed. Hence, the total number of remaining papers becomes 72. The remaining papers were checked against the title and abstract and only 44 papers were included. The remaining papers were checked against the inclusion and exclusion criteria (see Sect. 2.3), and 22 articles were found to meet the inclusion criteria. After which 4 papers were added to provide guide on review process and a total of 26 papers were included in the study to provide empirical evidence to address the research questions (see Sect. 2.1). The study search in this review study were conducted based on the Preferred Reporting Items for Systematic Reviews and Meta-Analysis (PRISMA) as seen in Fig. 1.

### Inclusion and Exclusion Criteria

The key terms are searched and retrieved articles were selected and included for this study based on the title and abstract information. The paper selection criteria include papers written in English language, published and related to the research questions (see Sect. 3.1). Studies not related to the research question or written in other languages are excluded from the review. Lastly, to ensure rigor and quality all selected studies are indexed in Scopus and Web of Science database.

## Findings

### Significance of Telemedicine Health Consultation to Manage Covid-19 Pandemic

Telemedicine refers to the utilization of modern Information and Communication Technologies (ICT) to deploy a network-based information platform that connects medical institutions, medical professionals, and patients remotely [15]. Telemedicine facilitates provision of healthcare delivery and has rapidly become a necessary tool that provides continuity of healthcare amidst pandemic [16]. Furthermore, telemedicine facilitates effective way to triage and provide timely, quality healthcare during public health emergency. It ensures the continuity of healthcare for outpatients [4], support medical professional on the front-line, and reduce the spread of COVID-19 infection while supporting social distancing [1]. Findings from Bray et al. [17] suggest that telemedicine integration has streamline clinic efficiency and helped in reaching patients remotely and patient were overwhelmingly positive in using telemedicine for treatment.

Additionally, telemedicine is integrated to help prevent healthcare systems from becoming overburdened and preventing medical professional burnouts [16]. Although, telemedicine excludes the face to face examination of patients, it allows collection of medical information before patient's admission and is useful for preoperative evaluation [5]. It can also be used for triaging and screening of patients with established or suspected infection, thereby protecting physicians and other patients [5]. Telemedicine is an important treatment option for patients who do not need urgent treatment. It provides information on the patient`s symptoms progression, thus helping identify acutely ill patients [5]. While, telemedicine cannot replace physical treatment, it can support in identifying patients at risk by providing information for further treatment and aids decision-making of attending physician [5]. It has the potential to reduce travel for patients who live far away. Thus, it decreases in-office hospital visits [18].

### Issues of Deploying Telemedicine Health Consultation

The integration of telemedicine in healthcare which may be synchronous or asynchronous involving audio or/and visual communications technology is faced with a few issues. Among these issues is the fact that telemedicine health consultation requires room staging, appropriate documentation, time management, and online rapport and engagement between the physician and patient. But not every physician or patient may be comfortable with integrating telemedicine platforms due to lack of physical social interaction and other resources required [6]. Furthermore, virtual or remote health care approaches in which the patients are geographically separated from the attending physician enabled by ICT have existed for decades, but widespread integration has been inhibited by policies, laws, and regulations at federal, state, and organizational levels [4]. This is because most national and commercial payers did not cover telemedicine services [6].

Accordingly, existing polices and regulations that do not support the integration of telemedicine in some countries. However, in the United States (US) on March 17, 2020, the Centers for Medicare & Medicaid Services (CMS) issued a waiver and expanded telemedicine coverage for all Medicare patients during the COVID-19 pandemic [18]. The change in CMS’s policy eliminate the main barriers to telemedicine integration which addressed other issues such as licensing restrictions, the lack of reimbursement, and Health Insurance Portability and Accountability Act (HIPAA) compliance [1, 16, 17]. Additionally, there is lack of training for medical professional on the use of telemedicine [5]. But, due to change in CMS policies findings from Bray et al. [11] reveal that their institution is now providing rapid online training for the practice of telemedicine in accordance with CMS, industry, and Emory University guidelines to train medical practitioners which became certified within few days.

Moreover, there is lack of security and inadequate technological infrastructures such as broadband connection and platforms for both medical professionals and patients [5, 19]. Due to the novelty and potentially unclear nature of telemedicine platforms to patients. There is lack of training to educate patients at least 15 min prior to their health consultation on telemedicine tools regarding how to check-in or login, etc.[18]. In addition, a recent telemedicine consumer research in the US observed that while up to 66 percent of patients were ready to utilize telemedicine in 2019, only 8 percent had utilized it previously. Mihalj et al. [5] postulated that the main issues include lack of patient understanding of new technology and lack of trust towards consulting physician whom they have never met in person. Findings reported by Mihalj et al. [5] indicated that majority of patients would prefer to consult their personal physician instead of using telemedicine for treatment.

### Recommendations for Deploying Telemedicine Health Consultation

Due to the COVID-19 pandemic, countries were placed under lockdown in order to manage and mitigate spread of COVID-19. To this end medical centers have reduced physical health consultation to telemedicine health consultation in response to the coronavirus pandemic [2]. Evidently, telemedicine offers several benefits for healthcare delivery, including providing expanded healthcare access to patients, and enabling more efficient healthcare management [18]. For telehealth to be successfully integrated, several berries such as availability of broadband coverage and internet access, licensure, reimbursement, and data security and privacy needs to be adequately addressed [20]. Thus, secured software platforms that conforms to HIPPAA standards or according to the standards set by national agencies should be adhered to ensure that patients medical data are secured from cyber security attack [5, 19].

Moreover, regulations and policies should include payment of medical professionals that deliver healthcare using traditional healthcare as well as telemedicine healthcare via online consultation, or telephone treatment [6]. However, telemedicine practices should be thoroughly review for payer’s policies to determine applicable telemedicine payments. Thus, the development of telemedicine-based fee schedule to help coding and reimbursement implications across payers should be provided for administration and providers [6]. The current workflow of medical professional needs to be updated in order to effectively integrate telemedicine services. Medical professional needs to support the re-scheduling and usage of technology such as electronic medical records with telemedicine applications to connect physician and patients [6]. Depending on the size of the medical center and availability of medical professionals it may be required to rotate medical professionals’ physical activities in the hospital and telemedical health consultations concurrently [6].

Accordingly, to enhance current telemedicine capabilities in hospital the provision of scalable and reliable infrastructure that can be embedded within the normal medical operations after the COVID-19 crisis is preferred to be deployed [6]. Moreover, it is important that patients and medical professionals both understand the significance of protecting confidentiality and privacy [1]. This can be accomplished by accurately encrypting personal health information transmitted via email. Also, physicians should always ask of patient consent before beginning the telephone or virtual health consultation [3]. Lastly, patients should be educated on the security, privacy, and personal benefit of using telemedicine [15]. Instructional resources should be communicated to patients and physicians [5].

### Software-Defined Networking to the Rescue

Software-defined networking (SDN) has the potential to address the technological issues, including the security and privacy concerns that may arise in employing telemedicine health consultations [13]. For example, AT&T observes that SDN enabled it to accommodate the sudden surge in the demand for Internet traffic during the lockdown imposed in several parts of the world due to COVID-19. This is as a result of the scalability and the dynamic nature of SDN that allows additional capacity to the network to be added in response to the increased traffic which before now is practically impossible to achieve. In the same way, SDN can be deployed to meet the unprecedented growth in the number of telemedicine health consultations triggered due to the ongoing global health crisis [14].

SDN is a current network paradigm in which the control plane is separated from the data plane [21]. In contrast to the existing network architecture where the control and data plane are part of the network devices, roles are separated in SDN such that the network devices can become purely forwarding devices with the forwarding instructions issued to them by the control plane and allowing the use of vendor-neutral components [22]. In SDN, the main objectives are to simplify the deployment of control plane functions and to support the applications to deal with a single abstracted network environment without regards for the implementation details, in addition to lowering the dependency on proprietary components [10, 22]. Therefore, SDN enables network functions to be directly programmable, which facilitates the automation of network functions and the building of highly scalable and flexible networks that can quickly adapt to changing environments [14].

**Fig. 2:**
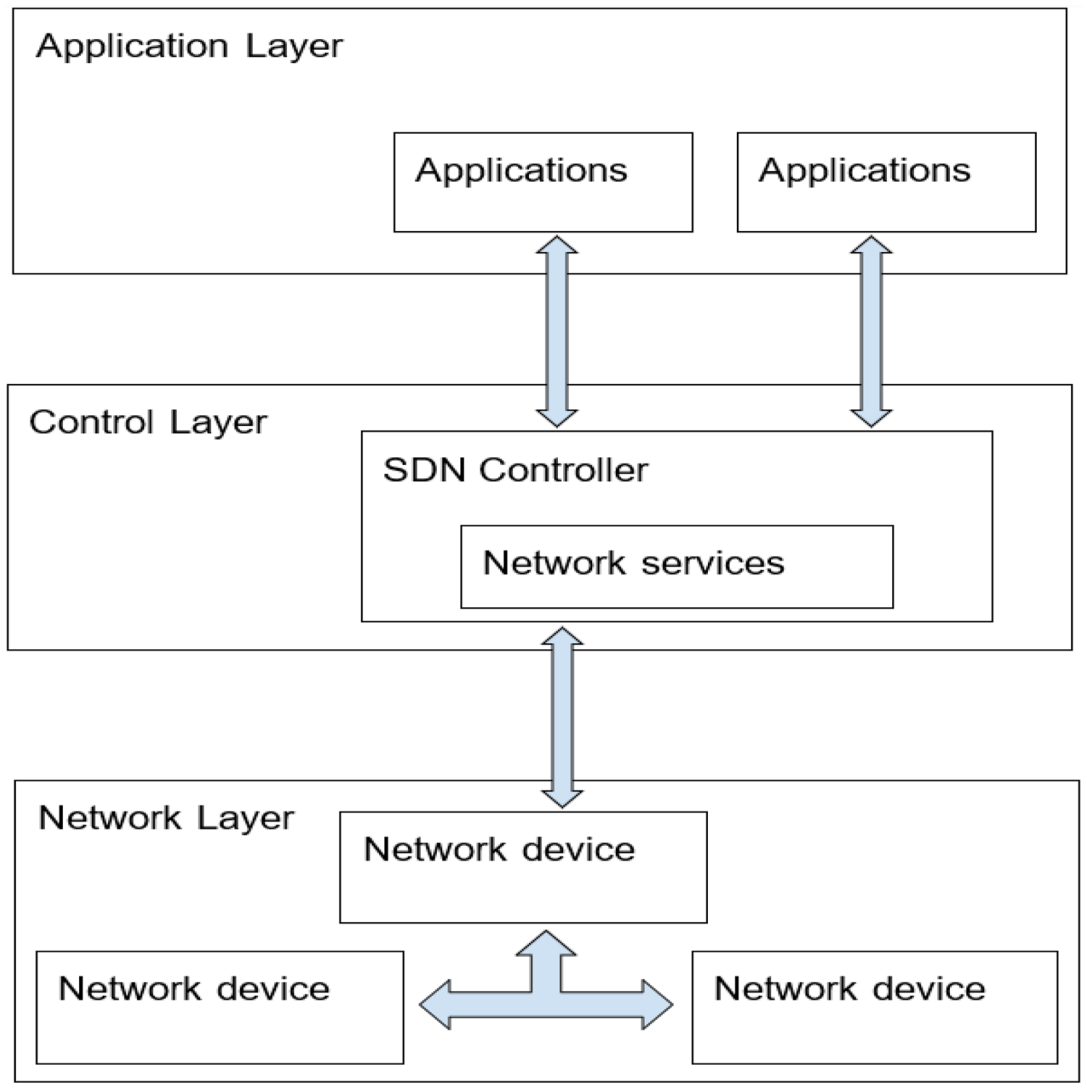
A typical SDN architecture

The SDN architecture is depicted in Fig. 2 and it consists of the application layer, control layer, and the network layer. The application layer is responsible for sending the applications’ requests for the network to the control layer, and this interaction is accomplished via the northbound interface. The control layer translates applications’ requirements and exercise control over the network devices, while providing the required information to the application. Also, the network layer is made up of network devices which interact with the control layer via the southbound interface. The implication of this setup is that the network intelligence is logically centralized in the SDN controller, which provides increased visibility into the entire network. Hence, the network would appear to the applications and policy engines as a single, logical switch. Therefore, the network devices no longer need to store and process several protocols but rather, accept the instructions forwarded to them by the SDN controllers.

Moreover, telemedicine health consultations rely on a robust network architecture to ensure effective and efficient communication between medical professionals and outpatients. The sudden surge in the demand for telemedicine due to the ongoing global crisis is already straining the existing network infrastructure. Many of the existing systems have legacy networks usually managed individually. It is usually not possible to dynamically scale these networks to meet the increase in network traffic. However, SDN offers an alternative network architecture which is able to help telemedicine providers to dynamically scale their network infrastructure in response to the service demand, reduce the complexity in managing the network, enhance security through improved visibility into the network, decrease the cost of network management, and simplifies compliance to the relevant legal and regulatory requirements. Thus, in this current study we propose in the following subsection SDN-based telemedicine architecture which can be used to meet the growing demand for telemedicine health consultations.

### Proposed SDN-based Telemedicine Architecture

**Fig. 3:**
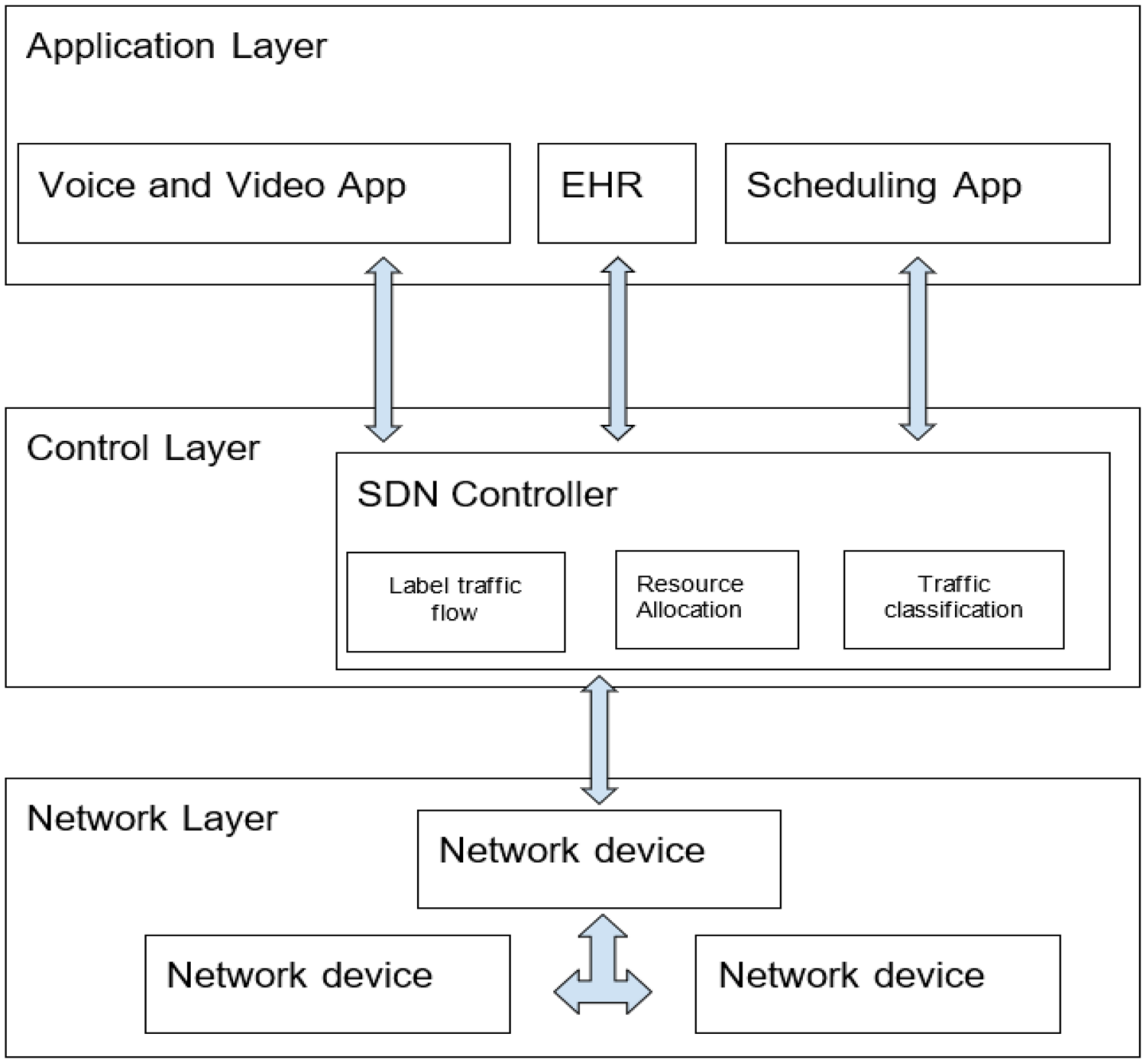
Proposed SDN-based telemedicine architecture

To address the need for telemedicine health consultations especially during this COVID-19 era, we propose SDN-based telemedicine architecture which takes into account its unique features. The proposed architecture is shown in Fig. 3.

Figure 3 depicts the proposed SDN-based telemedicine architecture which illustrates that the application layer consists of the applications used for telemedicine health consultation. Depending on the size and the resources of the telemedicine provider, the applications would include but not limited to real-time video and voice communication application, scheduling application, electronic health record, diagnostic viewer, payment gateway, and practice management application. These applications are able to communicate with the control layer via the northbound interface. Using this interface, the applications’ requests for network resources are sent to the SDN controller. Also, this architecture allows prioritization of traffic by ensuring that traffic for critical functions are handled first before non-critical functions. The labeling and assignment of network resources for critical functions is processed at the control layer.

The control layer translates the requirements of the applications and exert control over the network devices. It includes among other capabilities, functions for labeling traffic flow, resource allocation and traffic classification. The labeling of traffic is used to identify the type of traffic flowing through the network which is then employed during resource allocation and traffic classification. Resources are allocated based on the type of traffic. For example, in the case of telemedicine, voice and video are usually the traffic with the highest priority. Hence, with resource allocation and traffic classification, the SDN controller would ensure that voice and video traffic are transmitted without delay over other types of traffic. These instructions on how to handle the different types of traffic in the network are then pushed to the network devices by the SDN controller via the southbound interface.

In the network layer, we have network devices which are purely forwarding devices that forward the instructions pushed to them from the SDN controller. The network devices have a flow table where the instructions from the SDN controller are stored. When the network device receives a new flow, the SDN controller is contacted via the southbound interface for instruction on how to handle the flow. On receiving the flow, the SDN controller provides the needed information and pushes them back to the network device. The network device then forwards the flow based on the received instruction and updates its flow table with the same instruction. If the same flow is received again by the network device, it is able to forward the flow using the information in its flow table without consulting the SDN controller.

## Discussion and Implications

### Discussion

The term telemedicine refers method and technologies employed to deliver health care at a distance without direct physical contact among the physician and patient. Telemedicine encompasses both synchronous (audio and video) and asynchronous (e-consults, patient portal messages, through chatbots (virtual agents), and wearable devices). Telemedicine overcome physical barriers to provide patients and physician access to convenient healthcare while conserving personal protective equipment during pandemic [23, 26]. Findings from this study suggest that the current COVID-19 pandemic has stimulated regulators and payers to encourage integration of telemedicine during the health crises. Policy restrictions for telemedicine are temporarily lifted and virtual health consultation services are now currently been reimbursed. For example, Medicare patients are now been reimbursed for using telemedicine in place of traditional assessment regardless of patient location [6].

As suggested by Massaad and Cherfan [16], findings from this study highlights the need for policy changes to unleash the potentials of integrating telemedicine to improve health consultation. Moreover, findings from Wosik et al. [23] indicated that previously before the COVID-19 pandemic only 100 video health consultations were recorded daily. But presently a total of about > 600 patients are seen daily vial telemedicine video health consultations, with many in-person treatment replaced with telephone or video visits. Wosik et al. [23] pointed that in their medical institution, the number of telemedicine visits has increased within a month from below 1% total visits to a total of 70% visits, amounting to more than 1000 video health consultation per day. Additionally, findings from Bray et al. [11] suggested that they integrate online-based telemedicine video platform for outpatient health visits. This has led to the authors creating new workflows for virtual health delivery setting.

Similarly, findings from the literature [17] reveals that medical professionals are currently deploying telemedicine audio/visual platforms [24, 25], such as Zoom to communicate remotely. Further, findings from Greven et al. [18] confirm the usage of Zoom application as their preferred platform for delivering telemedicine due to its compliance with HIPAA and medical professional familiarity with the application. According to Greven et al. [18] Zoom is used as it provides features such as breakout rooms, which supports telemedicine visits which closely mimic the conventional workflow of physical hospital visit, with waiting room.

### Theoretical and Practical Implications

Evidently, telemedicine can help evaluate the presence of symptoms through close virtual observation of the patient's physical appearance. It supports early health information gathering that allows physician to strategically decide if physical treatment should be scheduled, thereby decreasing rates of exposure. It facilitates remote monitoring of blood pressure, blood sugar, electrocardiogram (ECG), and even a heart beats via electronic stethoscope. With this information, the physician can make recommendations for medications [5]. Currently, telemedicine is based on IP protocols which is widely employed for communication network. The IP-based networks are faced with a few limitations which make them difficult to achieve QoS during telemedicine. This is due to the fact that in IP-based networks, the data and control plane are not separated [9]. In this study SDN is applied to eliminate the limitations of the IP-based networks in addressing QoS faced in telemedicine similar to prior studies [9, 10, 14]. SDN is a network paradigm which separates the data and control plane.

This approach enables dynamic and fine-grained network configuration and management for telemedicine platforms to achieve QoS for patients and physicians during health consultations especially during the lockdown. Theoretically, this current study contributes to existing body of knowledge by applying SDN technology in order to achieve quality of service for telemedicine health consultation. The proposed SDN-based telemedicine architecture ensures the reliable transmission of medical data traffic between the physician and patients. The architecture can help to achieve proper transport protocols for improved QoS in reducing end-to-end delay, packet loss, and jitter during telemedicine health consultations [9]. The proposed architecture manage telemedicine traffics in an effective manner over SDN-based communication network to achieve QoS. This study provides practical implication on how to best integrate telemedicine into current healthcare by specifying the significance of telemedicine. Findings from this study helps in defining the issues faced, recommendation for integrating telemedicine and presenting an SDN telemedicine architecture to best achieve an optimal QoS telemedicine operations.

## Conclusion

Telemedicine support patients remotely to decrease physical hospital visits during health crises. Telemedicine aids the continued provision of personalized healthcare to patients in their homes using virtual technologies. Findings from the literature reveal that telemedicine improve access to care, supports monitoring and follow-up, and possibly reduce healthcare costs. This study utilizes secondary data from the literature to explore the application of SDN in improving telemedicine health consultation. Telemedicine uses video, audio, medical images, and data to provide remote healthcare services. Currently, due to many internet users there is decrease in data traffic [13]. Likewise, due to high packet loss and long end-to-end delay QoS impacts the effects on the performance of telemedicine platforms.

Providing QoS for telemedicine is one of the issues faced in communication networks [9]. Therefore, this study presents the significance of telemedicine, issues and recommendations of integrating telemedicine for health consultations. More importantly, this study proposes a telemedicine architecture to provide QoS for telemedicine platforms during health consultations. The proposed architecture employs SDN as a support network to provide QoS for telemedicine health consultations. This current study only employed secondary data from the literature. Hence, future work will involve conducting experiments and simulation to confirm the practically feasible of the proposed architecture for telemedicine to support QoS provisioning.
